# Abundant infiltration of B cells and plasma cells in brain biopsy of a male patient with severe anti-NMDA receptor encephalitis: A case report

**DOI:** 10.1097/MD.0000000000034237

**Published:** 2023-07-07

**Authors:** Linfei Wei, Zhengjuan Lu, Zunguo Du, Yin Wang, Hongzhi Guan

**Affiliations:** a Department of Neurology, Peking Union Medical College Hospital, Chinese Academy of Medical Sciences and Peking Union Medical College, Beijing, China; b Department of Neurology of Nanjing Drum Tower Hospital, Medical School and the State Key Laboratory of Pharmaceutical Biotechnology, Nanjing University, Nanjing, China; c Division of Neuropathology, Department of Pathology, Huashan Hospital of Fudan University, Shanghai, China.

**Keywords:** anti-NMDAR encephalitis, autoimmune, case report, immunopathology, male patient, pathology

## Abstract

Anti-N-methyl-D-aspartate receptor (NMDAR) is the most common type of autoimmune encephalitis mediated by NMDAR antibodies. The pathological process remains unclear, especially in patients without tumors or infections. Autopsy and biopsy studies have rarely been reported because of the favorable prognosis. Pathological findings generally demonstrate mild-to-moderate inflammation. This case report presents severe anti-NMDAR encephalitis in a 43-year-old man without any identified triggers. The biopsy in this patient showed extensive inflammatory infiltration with evident B cell accumulation, which enriches the pathological study of male anti-NMDAR encephalitis patients without comorbidities.

**Patient concerns:**

A 43-year-old previously healthy man presented with new-onset seizures with recurrent jerks. The initial autoimmune antibody test with serum and cerebrospinal fluid yielded negative results. After ineffective treatment for viral encephalitis, based on the imaging results indicating the possibility of diffuse glioma, the patient underwent a brain biopsy in the right frontal lobe to rule out malignancy.

**Diagnosis:**

The immunohistochemical study showed extensive inflammatory cell infiltration, consistent with the pathological changes in encephalitis. Cerebrospinal fluid and serum samples were then retested and tested positive for IgG antibodies against NMDAR. Therefore, the patient was diagnosed with anti-NMDAR encephalitis.

**Interventions:**

The patient was administered intravenous immunoglobulin (0.4 g/kg/d for 5 days), intravenous methylprednisolone (1 g/d for 5 days, 500 mg/d for 5 days, subsequently reduced to oral administration), and intravenous cyclophosphamide cycles.

**Outcomes:**

The patient developed refractory epilepsy 6 weeks later and required mechanical ventilation. Despite brief clinical improvement after extensive immunotherapy, the patient died from bradycardia and circulation.

**Lessons:**

Anti-NMDAR encephalitis cannot be ruled out even if the initial autoantibody test result is negative. For progressive encephalitis of unknown etiology, it is necessary to recheck cerebrospinal fluid for anti-NMDAR antibodies.

## 1. Introduction

Anti-N-methyl-D-aspartate receptor (NMDAR) encephalitis is the most common type of autoimmune encephalitis and is characterized by psychiatric symptoms, seizures, speech dysfunction, and movement disorders. A simplified model has been constructed for disease pathology in patients with combined tumors or viral infections as triggers.^[1]^ In contrast, the pathological process in patients without known triggers remains unclear, and such patients usually demonstrate severe outcomes, insensitivity to immunotherapy, and frequent relapses. Immunohistopathological studies with brain biopsies or autopsies are rarely reported because of the favorable prognosis. Previous pathological findings typically demonstrated mild or moderate inflammatory infiltration with variability owing to varied presentations and co-pathology (summarized by Zrzavy et al^[2]^). Here, we present the case of a male patient with severe anti-NMDAR encephalitis who was not identified with any associated disease. The brain biopsy demonstrated extensive inflammatory cell infiltration with predominant perivascular cuffing of B cells, partially supplementing the blank space in the pathological study of male anti-NMDAR encephalitis patients without triggers.

## 2. Case report

**Figure 1.**
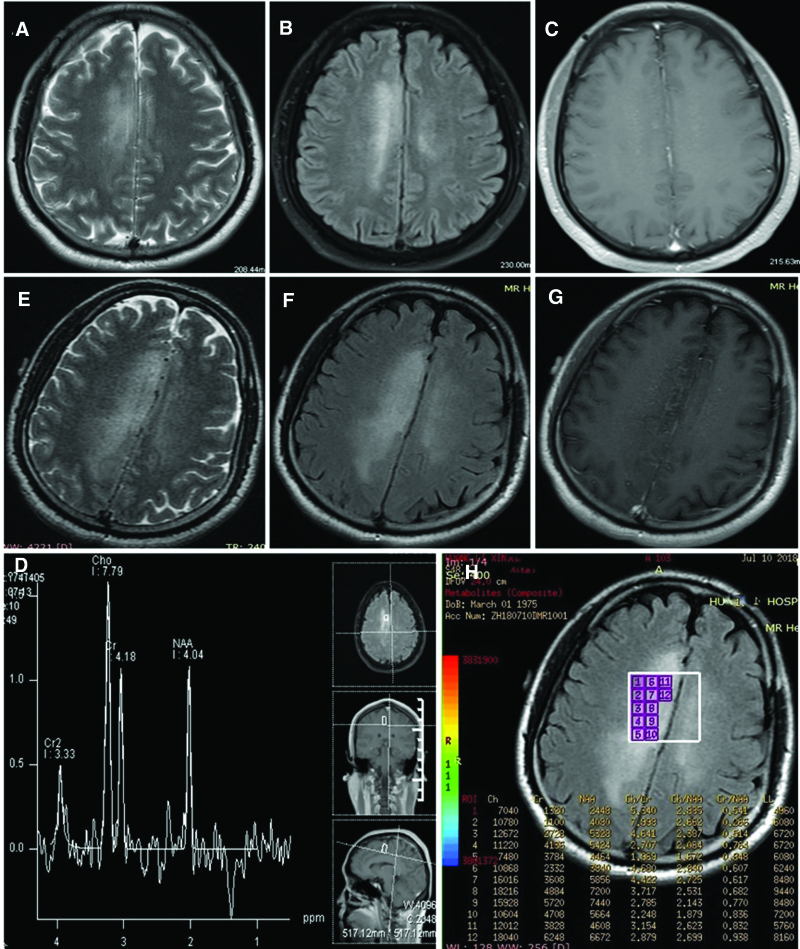
MRI obtained at 7 (A and B), 14 (C and D), and 21 (E–H) days after disease onset. (A and B) Axial T2 weighted and FLAIR images demonstrating a high signal intensity lesion in bilateral deep frontoparietal lobes (right > left). (C) Contrast-enhanced magnetic resonance image showing no enhancement of the lesion. (D and H) Magnetic resonance spectroscopy revealing an increased Cho peak, decreased NAA peak, and locally increased Lac peak. Cho/NAA ratio is 1.672–2.885. (E and F) Axial T2 weighted and FLAIR images revealing more extensive lesions in bilateral deep frontoparietal lobes. (G) Contrast-enhanced magnetic resonance image showing scattered small vessels and spot-like enhancement of the lesion. Cho/NAA = choline /N-acetyl aspartate, FLAIR = fluid attenuated inversion recovery, MRI = magnetic resonance imaging.

A 43-year-old previously healthy man was admitted to a local hospital in June 2018 for new-onset seizures with recurrent jerks on his left arm and left leg, lasting 2 to 3 seconds within a 30-minute interval. The results of general and neurological examinations were normal. Serum and cerebrospinal fluid (CSF) autoantibodies related to autoimmune encephalitis were negative at that time. T2 weighted and fluid attenuated inversion recovery hyperintensities were observed in the bilateral deep frontoparietal lobes on magnetic resonance imaging (MRI) 7 days after commencement (Fig. 1A and B). The patient was diagnosed with viral encephalitis and received acyclovir (1.5 g/d) and oral carbamazepine, however, no improvement was observed. Fourteen and twenty-one days after commencement, brain MRI (Fig. 1C–H) revealed more widespread lesions in the bilateral frontoparietal lobes, with scattered tiny vessels and spot-like enhancement. The results of MRI and magnetic resonance spectroscopy suggested the possibility of diffuse glioma.

**Figure 2.**
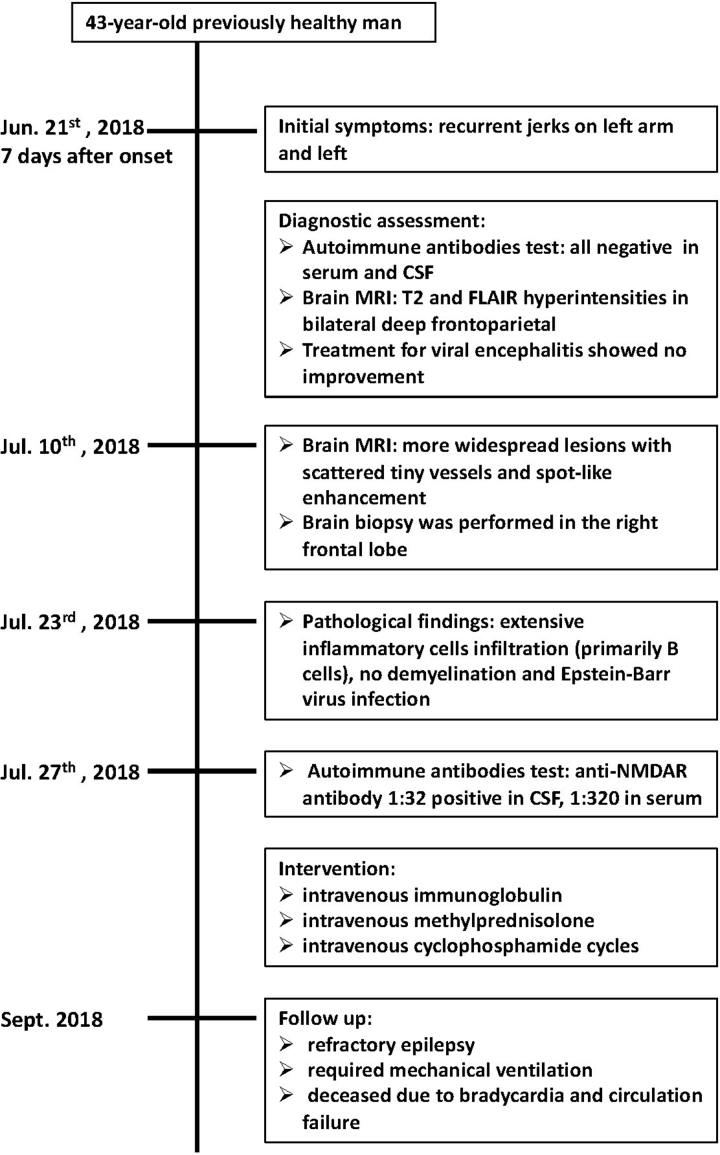
Timeline of diagnosis, interventions, and outcomes.

To rule out malignancy, a brain biopsy was performed in the right frontal lobe, obtaining a 3 × 1 cm broken brain tissue. The pathological findings of prominent lymphocytic inflammation supported the diagnosis of encephalitis. CSF and serum samples were retested and were positive for anti-NMDAR antibodies at 1:32 and 1:320. The patient was diagnosed with anti-NMDAR encephalitis and then administered intravenous immunoglobulin (0.4 g/kg/d for 5 days), intravenous methylprednisolone (1 g/d for 5 days, 500 mg/d for 5 days, subsequently reduced to oral administration), and monthly cyclophosphamide cycles. The patient developed refractory epilepsy and was admitted to critical care 6 weeks later, requiring mechanical ventilation. Although he experienced brief clinical improvement after extensive immunotherapy, he died due to bradycardia and circulatory failure (Fig. 2).

### 2.1. Brain biopsy

Hematoxylin-eosin staining, as well as immunohistochemical staining with antibodies against CD20 (primarily B cells), CD138 (primarily plasma cells), CD2 (primarily T cells), CD68 (primarily macrophages and activated microglia), and myelin basic protein, were performed on paraffin-embedded tissue, followed by appropriate biotinylated secondary antibodies. In situ hybridization of the Epstein-Barr encoding region was used to rule out Epstein-Barr virus infection.

**Figure 3.**
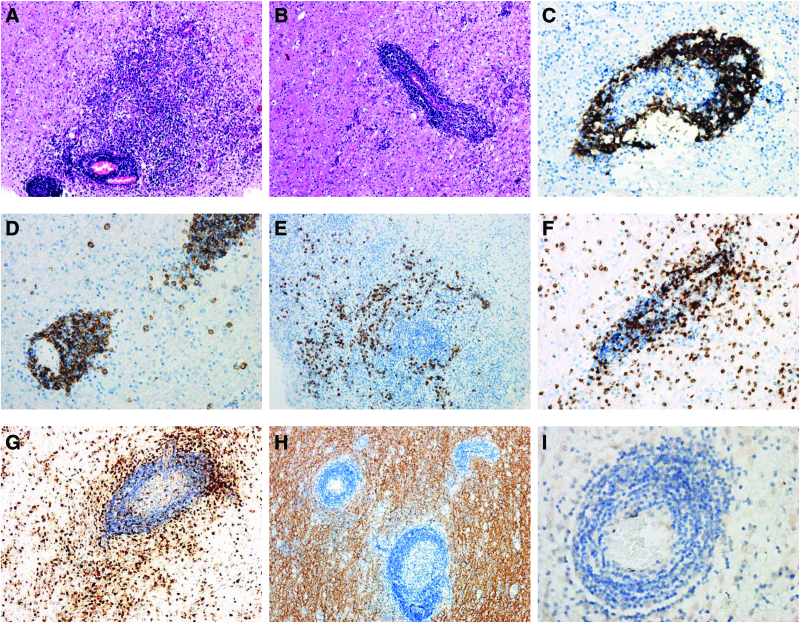
Neuropathological findings in the right frontal lesion of the patient. Hematoxylin-eosin staining (A and B). An immunohistochemical study using anti-CD20 (C), anti-CD138 (D and E), anti-CD2 (F), and anti-68 (G) antibodies. Immunoperoxidase staining. There are prominent perivascular cuffing of CD20^+^ B cells and CD138^+^ plasma cells, with plasma cells and CD2^+^ T cells spreading in the parenchyma. CD68 staining demonstrates extensive macrophages and activated microglia. Anti-MBP antibody staining (H) reveals no signs of demyelination. EBER in situ hybridization is negative (I). Magnification × 10 (A, B, E, G, and H) and × 40 (C, D, F, and I). EBER = Epstein-Barr encoding region, MBP = myelin basic protein.

The pathological findings included mild neuronal loss, microglia activation, and inflammatory changes, with lymphocytic infiltration observed in the perivascular space and brain parenchyma (Fig. 3). Prominent infiltration of CD20^+^ B cells (Fig. 3C) was found limited to the perivascular region, while CD138^+^ plasma cells displayed a more dispersed distribution with disbandment throughout the parenchyma (Fig. 3D and E). CD2 staining revealed moderate T cell infiltration around the vessels and parenchyma. CD68^+^ macrophages and activated microglia were present in the perivascular region and the parenchyma (Fig. 3G). Demyelination and Epstein-Barr virus infection were absent, according to the negative results of myelin basic protein immunostaining and in situ hybridization of the Epstein-Barr encoding region, respectively (Fig. 3H and I).

## 3. Discussion

This case report presents a patient with severe encephalitis without abnormalities in the initial serum and CSF examination, whose MRI indicated a high signal on T2 weighted and fluid attenuated inversion recovery in the bilateral deep frontoparietal lobes. Therefore, the patient was not diagnosed with anti-NMDAR encephalitis, but was considered viral encephalitis followed by diffuse glioma. We believe that this was not a false positive result in the second antibody detection because it was positive at high dilutions in both CSF and serum. We propose that it is necessary to reconfirm the CSF autoimmune antibody results, especially when clinical manifestations, imaging, and laboratory results cannot completely confirm or refute the diagnosis of autoimmune encephalitis.

The immunopathological results of the biopsy indicated predominant perivascular cuffing of CD20^+^ B cells and CD138^+^ plasma cells, which has been generally reported in the previous neuropathological studies (summarized by Zrzavy et al^[2]^), supporting the intrathecal synthesis of antibodies in anti-NMDAR encephalitis. CD68 staining shows abundant CD68^+^ macrophages and activated microglia, as seen in some previous neuropathological findings with severe courses of anti-NMDAR encephalitis.^[2–5]^ The relatively widely distributed CD68^+^ macrophages and activated microglia, and CD2^+^ T cells may be responsible for the severity of the inflammatory reaction. However, further research is required to illustrate the precise function of these inflammatory cells and to explain the entire pathological process.

## 4. Summary

The autoimmune mechanism of anti-NMDAR encephalitis is still unclear, particularly in patients without tumors. The prominent plasma cell and B cell accumulation, shown in this rare-reported immunohistopathological study of a male patient without associated disease, supports the theory of intrathecal autoantibody synthesis in anti-NMDAR encephalitis. The accumulation of T cells, macrophages, and activated microglia may be related to disease severity and refractoriness to immunotherapy.

## Author contributions

**Investigation:** Zhengjuan Lu, Zunguo Du, Yin Wang.

**Project administration:** Hong-Zhi Guan.

**Writing – original draft:** Linfei Wei.

**Writing – review & editing:** Zhengjuan Lu, Hong-Zhi Guan.
